# Identification of Isoxsuprine Hydrochloride as a Neuroprotectant in Ischemic Stroke through Cell-Based High-Throughput Screening

**DOI:** 10.1371/journal.pone.0096761

**Published:** 2014-05-07

**Authors:** Jeff W. Hill, Jeffrey F. Thompson, Mark B. Carter, Bruce S. Edwards, Larry A. Sklar, Gary A. Rosenberg

**Affiliations:** 1 University of New Mexico Health Sciences Center, Department of Neurology, Albuquerque, New Mexico, United States of America; 2 Center for Molecular Discovery, University of New Mexico Health Sciences Center, Albuquerque, New Mexico, United States of America; National University of Singapore, Singapore

## Abstract

Stroke is a leading cause of death and disability and treatment options are limited. A promising approach to accelerate the development of new therapeutics is the use of high-throughput screening of chemical libraries. Using a cell-based high-throughput oxygen-glucose deprivation (OGD) model, we evaluated 1,200 small molecules for repurposed application in stroke therapy. Isoxsuprine hydrochloride was identified as a potent neuroprotective compound in primary neurons exposed to OGD. Isoxsuprine, a β_2_-adrenergic agonist and NR2B subtype-selective N-methyl-D-aspartate (NMDA) receptor antagonist, demonstrated no loss of efficacy when administered up to an hour after reoxygenation in an in vitro stroke model. In an animal model of transient focal ischemia, isoxsuprine significantly reduced infarct volume compared to vehicle (137±18 mm^3^ versus 279±25 mm^3^, *p*<0.001). Isoxsuprine, a peripheral vasodilator, was FDA approved for the treatment of cerebrovascular insufficiency and peripheral vascular disease. Our demonstration of the significant and novel neuroprotective action of isoxsuprine hydrochloride in an in vivo stroke model and its history of human use suggest that isoxsuprine may be an ideal candidate for further investigation as a potential stroke therapeutic.

## Introduction

On average, every 40 seconds, someone in the United States has a stroke and every 4 minutes someone dies from a stroke [1]. Stroke accounted for 1 of every 19 deaths in 2009 and, when considered separately from other cardiovascular diseases, ranks number 4 among all causes of death [1]. Accordingly, there is much interest in therapeutic intervention to decrease neurological damage and prevent death after stroke. Despite the prevalence of stroke, currently, the only FDA-approved drug for the treatment of stroke is the thrombolytic agent recombinant tissue plasminogen activator (tPA) [2]. tPA use is severely limited by the need to use the drug within 4.5 hours after stroke to be effective. Even if used within the therapeutic window, tPA has a risk of symptomatic intracerebral hemorrhage [2]. New treatment options are critically needed to extend the therapeutic window for thrombolysis and provide neuroprotection to slow cell death after stroke.

Currently, it takes an average of 15 years and $800 million dollars to bring a new drug to market [3]. Each year, the FDA approves 20–30 new drugs for human use. At that rate, it will take approximately 300 years for the number of drugs currently in use to double. It follows that the repurposing of existing drugs for novel applications may represent a more fruitful approach to drug discovery than the discovery of a new drug. The use of high-throughput screening for the repurposing of characterized agents with a history of human use is an important part of the National Center for Advancing Translational Sciences roadmap to enhance the path from drug to patient [4].

To date, two high-throughput screening studies for novel stroke drugs have been reported. In one study, organotypic rat brain slices were used to evaluate the effects of small molecules on neuronal survival in an OGD stroke model. The study identified neriifolin, a cardiac glycoside, as a candidate stroke drug in the OGD model and confirmed in vivo functionality of the molecule in an animal model of stroke [5]. In another study, a combination of primary neurons and hippocampal brain slices were used to screen a chemical library for neuroprotective compounds. The study identified carbenoxolone as a neuroprotectant and demonstrated neuroprotection by the compound in an animal stroke model [6].

Here, we report the identification of isoxsuprine hydrochloride as a potent neuroprotectant against ischemia via cell-based high-throughput screening. Isoxsuprine was identified as a top neuroprotectant among 1,200 compounds in the Prestwick Chemical Library, which contains diverse, mostly FDA-approved and off-patent compounds, and its neuroprotective function was confirmed in transient ischemic injury in animals.

## Materials and Methods

### Primary cortical neuron culture

Cortical neurons were obtained from Spraque-Dawley rat embryos at day 18 of gestation. Cortices were triturated and digested with 0.025% trypsin in phosphate buffer saline (PBS) for 10 minutes. Digestion was stopped by the addition of Dulbecco's Modified Eagle Medium (DMEM) containing 10% fetal bovine serum (FBS). Trypsinized cells were filtered using 70 µM disposable cell filters (Becton-Dickinson), washed twice with Neurobasal medium (Gibco) containing 4% FBS, and preplated 4 times for 30 minutes in 150 mm vacuum-plasma treated dishes (Becton-Dickinson). During these preplating steps, primarily non-neuronal cells adhere to the dish, thereby enriching the neuronal population. Enriched neurons were plated at a density of 50×10^3^/cm^2^ in Neurobasal medium containing 4% FBS, 0.5 mM Glutamax (Gibco), and penicillin-streptomycin (Gibco) in white clear-bottomed 96-well plates (353377) or 24-well plates (353226) (Becton-Dickinson) coated overnight with 50 µg/ml poly-D-lysine. At plating, spaces between wells in 96-well plates were filled with 50 µl sterile water to minimize evaporation of medium at the edges of the dish. Twenty-four hours later (1 day in vitro, 1 DIV), 50% of the medium was replaced with Neurobasal medium, 2% B27 (Gibco), and 4 µM cytosine arabinoside (AraC) (2 µM AraC final). At 5 DIV, 50% of the medium was replaced with Neurobasal, 2% B27. At 7 DIV, cortical cultures were 98% neurons as assessed by microtubule-associated protein 2 (MAP2) antibody labeling and flow cytometry analysis (data not shown).

### High-throughput oxygen-glucose deprivation model of stroke

The Prestwick Chemical Library (Prestwick Chemical) was screened for neuroprotection in OGD by pretreating 7 DIV cortical neurons in 120 µl medium in 96-well dishes with 10 µM compounds for 30 minutes. All liquid handling was automated and performed by a Beckman Coulter Biomek NX multichannel pipettor to eliminate potential manual pipetting bias. The pretreatment medium was transferred to a clean plate for storage and was kept at 37°C in a 5% CO_2_ environment during the OGD exposure. After pretreatment, cells were washed 3 times with 200 µl deoxygenated glucose-free Hank's buffered saline solution (HBSS), pH 7.4, to remove glucose and oxygen and were covered with 100 µl deoxygenated glucose-free HBSS. HBSS was deoxygenated in a gas washing bottle with 60 liters of 95% nitrogen, 5% CO_2_ per 250 ml HBSS. HBSS was maintained at 37°C during deoxygenation in a water bath. Compounds were added to wells and plates were sealed with sealing tape and placed in a 37°C incubator for 2 hours (OGD). Eight wells per dish were used as dimethyl sulfoxide (DMSO)-treated vehicle-only controls. After 2 hours, HBSS was removed and 100 µl pretreatment medium was returned to the wells. After 24 hours, neuronal death (dead/live cell protease ratio) was quantified using CytoTox-Glo reagents (Promega) and a Victor^3^ V luminometer (Perkin Elmer) according to the manufacturer's instructions. Z-scores were calculated as previously described [7]. Compounds producing a z-score of at least 3 were considered hits [7] and underwent secondary screening.

### Secondary screening of primary hits

Neuroprotection by candidate compounds identified in the primary screen was assayed by measurement of apoptosis using terminal deoxynucleotidyl transferase dUTP nick end labeling (TUNEL) reagents as described previously [8]. At 7 DIV, primary neurons grown on poly-D-lysine-coated 22 mm-diameter cover slips in 24-well plates underwent 2 hours of OGD and were treated with a range of doses of each compound at reoxygenation. Compounds providing greater than 2-fold protection over vehicle were then administered at the optimal dose after OGD at intervals of 0, 15, 30, and 60 minutes post-reoxygenation. In vitro experiments were performed in quadruplicate and results are shown as means with standard deviation.

### Middle cerebral artery occlusion (MCAO) model

All animal procedures were approved by the University of New Mexico Institutional Animal Care and Use Committee and were in compliance with federal guidelines. Male spontaneously hypertensive rats (SHR) weighing 290–300 g were obtained from Charles River Laboratories. Animals were anesthetized with 2% isoflurane inhalant during all surgical procedures. The right common carotid artery (CCA) was ligated approximately 6 mm caudal to the bifurcation of the external and internal carotid arteries. With a vascular clamp in place on the CCA immediately caudal to the bifurcation, a punctate incision was made in the CCA 5 mm caudal to the clamp and a 4-0 nylon suture (Doccol Corporation) 30 mm in length with a 2–3 mm silicone coated tip (0.39 mm diameter) was advanced into the CCA lumen and secured in place with 2 4-0 silk sutures spaced 3 mm apart and tied around the CCA caudal to the clamp. The clamp was removed and the suture advanced into the internal carotid artery until resistance was felt. In this position, the filament occludes the middle cerebral artery (MCA) origin and MCA territory becomes ischemic. The suture was secured with the 2 silk sutures around the CCA. The wound was closed and the animal was allowed to recover during the 90-minute occlusion period. After 90 minutes, the suture was removed and the CCA ligated rostral to the incision. At reperfusion, animals received 0.5 ml of vehicle (0.6% DMSO in normal saline) or 1 mg/kg isoxsuprine hydrochloride by intravenous (IV) injection through the lateral tail vein. All animals received 3 ml of subcutaneous saline after surgery to prevent dehydration. After 24 hours reperfusion, animals were sacrificed, brains were sectioned into 4 mm-thick quadrants, and infarcted tissue was identified by 2,3,5-triphenyltetrazolium chloride (TTC) staining. Edema-corrected infarct volume was calculated by subtracting the area of non-infarcted tissue in the ipsilateral hemisphere from the total volume of the contralateral hemisphere. Infarct volume was quantified using Image J software.

### Statistical analyses

Statistically significant differences between in vitro drug treatment groups were determined by one-way analysis of variance (ANOVA) with Bonferroni's multiple comparison test. Comparison of infarct volumes between animals receiving vehicle or isoxsuprine hydrochloride was performed using a two-tailed unpaired t-test. Differences between experimental groups were considered statistically significant if *p*<0.05 with a 95% confidence interval.

## Results

### High-throughput screening

We identified 16 compounds with a z-score of 3 or greater. Of these 16 compounds, 10 compounds have not been previously reported to have efficacy in animal models of ischemia and underwent secondary screening (Table 1).

**Table 1:** Neuroprotective compounds identified through high-throughput screening of the Prestwick Chemical Library.

Z-score	Chemical name	Therapeutic group	Mechanism of action
5.567	*Dizocilpine maleate (MK-801)* 1	*-*	*Probe for NMDA receptors*
5.130	Mianserine hydrochloride	Antidepressant	5-HT antagonist
4.000	Isoxsuprine hydrochloride	Vasodilator	β-adrenergic agonist
3.800	Meropenem	Antibacterial	Peptidoglycan synthesis inhibitor
3.647	Meclofenamic acid	Anti-inflammatory	Cyclooxygenase inhibitor
3.540	*Guanabenz acetate*	*Antihypertensor*	*Alpha agonist*
3.428	*Candesartan*	*Antihypertensive*	*Angiotensin II receptor antagonist*
3.400	Etilefrine hydrochloride	Vasoconstrictor	Sympathomimetic
3.270	Haloperidol	Antipsychotic	Dopamine antagonist
3.200	Moxonidine	Antihypertensive	Imidazoline receptor agonist
3.166	*Bumetanide*	*Diuretic*	*Vascular cyclooxygenase activator*
3.142	*Ibudilast*	*Anti-inflammatory*	*Phosphodiesterase inhibitor*
3.117	Chlorphenesin carbamate	Muscle relaxant	-
3.000	*Chicago sky blue 6B*	*-*	*Competitive glutamate uptake inhibitor*
3.000	Prothionamide	Antibacterial (tuberculostatic)	-
3.000	Epitiostanol	Antineoplastic	-

1 Compounds previously investigated in models of cerebral ischemia are italicized.

### Secondary screening

**Figure 1:**
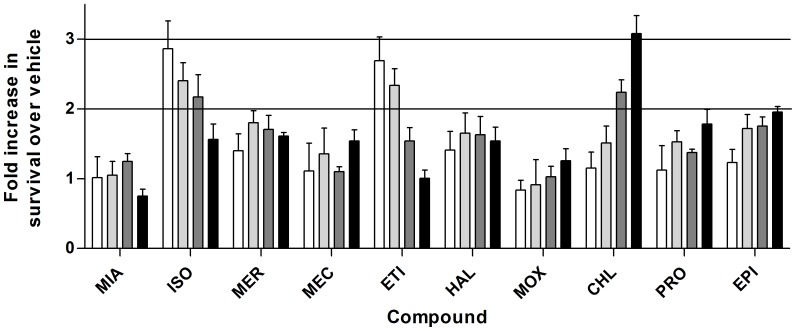
Secondary screening of neuroprotective compounds. Compounds identified through high-throughput screening were administered at 0.1 (white), 1 (light gray), 10 (dark gray), and 100 µM (black) at reoxygenation after 2 hours OGD. Twenty-four hours later, cells survival was measured by TUNEL assay and compared to DMSO vehicle-treated neurons. Compounds providing at least 2-fold increased neuroprotection over vehicle were further investigated. MIA, mianserine hydrochloride, ISO, isoxsuprine hydrochloride, MER, meropenem, MEC, meclofenamic acid, ETI, etilifrine hydrochloride, HAL, haloperidol, MOX, moxonidine, CHL, chlorphenesin carbamate, PRO, prothionamide, EPI, epitiostanol.

**Figure 2:**
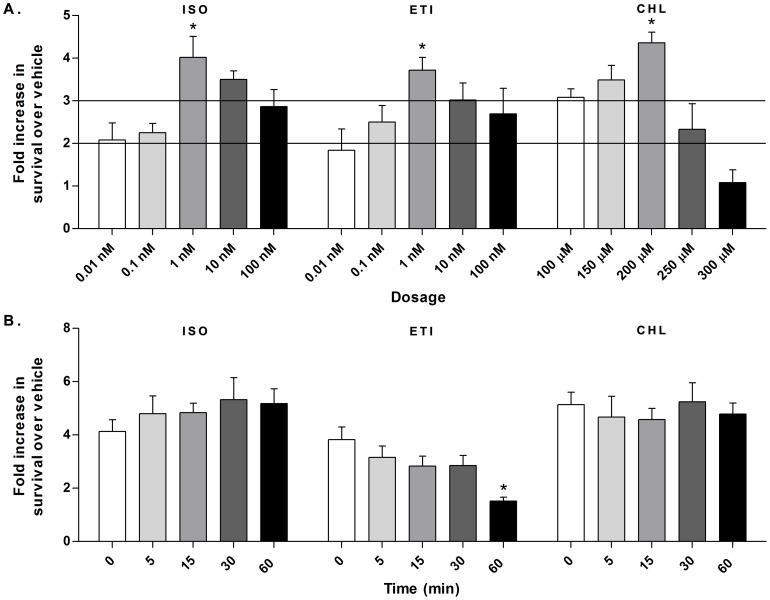
Dose optimization and time course of administration of neuroprotective compounds. A. Dose response of compounds administered at reoxygenation after 2(ISO) and etilifrine hydrochloride (ETI) and 200 µM for chlorphenesin carbamate (CHL). Isoxsuprine was significantly more protective at 1 nM compared to 0.01, 0.1, and 100 nM (*, *p*<0.01). Etilifrine was significantly more protective at 1 nM compared to 0.01 and 0.1 nM (*, *p*<0.05). Chlorphenesin carbamate was significantly more neuroprotective at 200 µM compared to all other doses (*, *p*<0.05). B. Time course of administration of compounds at the optimal dose at 0, 15, 30, and 60 minutes after reoxygenation onset. Isoxsuprine and chlorphenesin carbamate demonstrated no decrease in neuroprotection when administered up to 60 minutes after reoxygenation onset. Neuroprotection by etilifrine significantly decreased when administered at 60 minutes versus time 0 (*, *p*<0.01).

The neuroprotective function of the 10 compounds identified in the high-throughput screening OGD model was evaluated using the TUNEL assay, a direct measure of apoptosis. In the secondary screen, compounds were administered at reoxygenation in doses ranging from 0.1 to 100 µM. Compounds providing at least a 2-fold increase in survival over vehicle included isoxsuprine hydrochloride, etilifrine hydrochloride, and chlorphenesin carbamate (Figure 1). The optimal dose for these 3 compounds was determined by treating neurons with 0.01 to 100 nM compound for isoxsuprine and etilifrine and 100 to 300 µM compound for chlorphenesin carbamate at reoxygenation. The doses providing maximum neuroprotection against OGD-induced apoptosis were 1 nM for isoxsuprine and etilifrine and 200 µM for chlorphenesin carbamate (Figure 2 A). Of particular interest was determining if any of these active compounds could mediate neuroprotective effects when administered after reoxygenation. Using the optimal dose, compounds were then administered to neurons 0, 15, 30, and 60 minutes after the onset of reoxygenation after 2 hours of OGD. Isoxsuprine and chlorphenesin carbamate demonstrated no loss of neuroprotection when administered up to an hour after reoxygenation while a significant decrease in neuroprotection by etilifrine was observed with treatment at 60 minutes (Figure 2 B).

### Isoxsuprine in an animal stroke model

**Figure 3:**
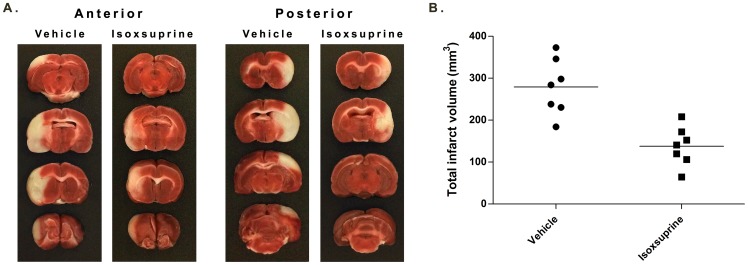
Neuroprotection by isoxsuprine hydrochloride in an animal stroke model. A. Representative TTC-stained brain sections showing differences in infarction between animals receiving vehicle or isoxsuprine hydrochloride. B. Effect of isoxsuprine hydrochloride on infarct volume. Isoxsuprine hydrochloride (1 mg/kg, IV) given at the onset of reperfusion after a 90-minute MCAO significantly reduced infarct volume compared to vehicle (137±18 mm^3^ versus 279±25 mm^3^), *p*<0.001. Closed circles, vehicle, closed squares, isoxsuprine hydrochloride, n = 7 animals for each group.

Although isoxsuprine and chlorphenesin carbamate were both neuroprotective within an extended therapeutic window in vitro, the optimal dose for chlorphenesin carbamate was 200 µM versus 1 nM for isoxsuprine. Thus, an effective dose of chlorphenesin carbamate may be difficult to attain or exhibit toxicity in vivo. To evaluate neuroprotection by isoxsuprine in vivo, an exploratory dose of 1 mg/kg isoxsuprine was administered by IV injection at reperfusion after a 90-minute occlusion of the right middle cerebral artery. Twenty-four hours after reperfusion onset, total edema-corrected infarct volume in the right hemisphere was calculated and compared between vehicle- and isoxsuprine-treated animals. Total infarct volume in vehicle-treated animals was 279±25 mm^3^ compared to 137±18 mm^3^ in isoxsuprine-treated animals (Figure 3).

## Discussion

The goal of this study was to utilize a cell-based high-throughput screening model to facilitate the identification of drugs with a history of human use which may have novel therapeutic uses in the treatment of acute ischemic stroke. Utilizing our high-throughput OGD stroke model, we identified isoxsuprine hydrochloride as a neuroprotectant and demonstrated efficacy of the molecule in stroke in animals.

The OGD in vitro ischemia model has been used extensively in the identification and evaluation of potential stroke therapeutics. The ease of use and reproducibility of the system, along with a low cost of implementation, contribute to its being amenable to preliminary experiments aiming to demonstrate biological activity of molecules before moving on to more costly and time consuming experiments in animal stroke models. OGD may be performed in organotypic brain slices, where the 3-dimensional arrangement of neurons, glial cells, and extracellular matrix is partially preserved, or in neuronal cultures which may consist of purified neurons, as in this study, or mixed populations of neurons, glial cells, and endothelial cells. While organotypic slices may most closely represent intact brain tissue, the method is labor- and time-intensive and expensive since numerous animals are required to manually prepare sufficient tissue sections needed to screen a chemical library which may contain thousands of compounds. Further, since multiple cell types are present, the establishment of neuron-specific cell death measures can be complex and complicates high-throughput screening efforts which typically benefit from rapid, easily implemented measures of cell survival.

In vivo, activation of glial cells and infiltration of immune cells at infarct sites during ischemia/reperfusion is believed to facilitate neuronal death. To approximate this situation, neurons may be co-cultured with astrocytes or microglia and subjected to OGD. In this mixed culture model, compounds that are neuroprotective may exert their effects through glial cells or directly on neurons. This latter possibility may be verified by comparing neuroprotection by a compound during OGD in mixed cultures and in pure neurons. To examine direct neuroprotection by a compound, a pure neuronal population may be used. Use of an enriched neuronal population eliminates possible confounding effects introduced by the presence of glial cells, such as astrocytes and microglia, which are typically activated ex vivo and may influence neuronal survival and responses to stressors in ways not reflective of an in vivo ischemia/reperfusion scenario. Additionally, the primary neurons obtained from the embryonic pups of a single animal can be sufficient to screen an entire chemical library. Further, since only one cell type is present in enriched neurons, any cell death observed can be specifically attributed to neurons without performing additional experiments. These latter advantages particularly favor the use of pure neuronal populations in streamlined preliminary high-throughput screening studies.

Our cell-based model was validated by the identification of MK-801 as the top hit in a high-throughput screen, along with 5 other compounds with previously reported efficacy in ischemic models. MK-801 is a potent neuroprotectant and the highest affinity NMDA receptor antagonist known. Although highly protective in animal and in vitro ischemia models, MK-801 has a limited therapeutic window and causes severe neurological side effects, including psychosis. Pretreatment with compounds was used to validate our assay and increase sensitivity in the high-throughput primary screen. As our results show, the number 2 hit, mianserine hydrochloride, was highly protective when used as a pretreatment but ineffective when given at reoxygenation in the secondary screen. Similar results were reported with MK-801, which was neuroprotective during OGD when used as a pretreatment but significantly decreased in effectiveness when administered as few as 5 minutes after the onset of reoxygenation [5]. Thus, while pretreatment with compounds is reasonable during high-throughput screening, a secondary screen with compounds given at or after reoxygenation in vitro or reperfusion in vivo should be employed as pretreatment scenarios may not be clinically relevant to the treatment of acute stroke. Additionally, as the measures of neuronal survival employed may significantly influence screening outcome, a secondary assay and direct measure of neuronal apoptosis such as TUNEL should be used to confirm neuroprotection observed in high-throughput assays.

Our identification of isoxsuprine hydrochloride, a well-characterized β_2_-adrenergic agonist and peripheral vasodilator, as a potent neuroprotectant in vitro and confirmation of its action in an animal stroke model both further validate the high-throughput screening assay and suggest a significant effect of adrenergic receptor activation in promoting neuronal survival in ischemic stroke. This latter possibility is supported by several studies demonstrating neuroprotection by β-agonists in animal and in vitro models of ischemia [9]–[11]. Clenbuterol, a β_2_-adrenoreceptor agonist, is protective during OGD in mixed neuronal cultures and in animal stroke models. Clenbuterol is believed to exert neuroprotection by activating astrocytes and stimulating the production of nerve growth factors (NGFs) which promote neuronal survival [10]. Interestingly, clenbuterol is present in the Prestwick Chemical Library but was not identified as a neuroprotective compound in our high-throughput screening assay utilizing pure cortical neurons (z-score of 0). This is consistent with the reported effects of clenbuterol on astrocyte-mediated NGF production. Thus, whether the “hits” emerging from a cell-based high-throughput screen target neurons, glial cells, or both may depend highly upon the in vitro OGD model utilized. While there is clear and significant involvement of adrenergic receptor activation in neuronal survival decisions in ischemic injury, the underlying mechanisms remain to be further characterized.

In an animal stroke model, co-administration of clenbuterol and memantine, an NMDA receptor antagonist, was shown to be more effective than administration of either compound individually, extended the therapeutic window for clenbuterol treatment, and has been proposed to be a potential clinical approach to stroke therapy [12]. Ifenprodil, an NR2B subtype-selective NMDA receptor antagonist and known neuroprotectant in stroke models [13], was neuroprotective in our primary screen but was just below the cutoff for being considered a hit (z-score of 2.916). The remarkable neuroprotection provided by isoxsuprine hydrochloride in the MCAO model may be related to the fact that isoxsuprine possess both β_2_-adrenoreceptor agonist [14] and NR2B subtype-selective NMDA receptor antagonist [15] functions in a single molecule. Isoxsuprine also possesses significant α_1_-adrenoreceptor antagonist activity [16], [17] which may further promote neuroprotection by enhancing β_2_-agonism-mediated cerebral vasodilation and inhibiting vasoconstriction in ischemic injury. Thus, isoxsuprine may synergistically exploit at least two known neuroprotective pathways while simultaneously increasing circulation to the fragile penumbral region after ischemia and preventing evolution of the infarct core.

## Conclusions

Our results demonstrate the identification of a potential stroke therapeutic through cell-based high-throughput screening of a chemical library. Isoxsuprine is of particular interest as a stroke therapeutic because of its extensive history of use in humans for the treatment of numerous indications, including cerebrovascular insufficiency, hypertension, Raynaud's phenomenon, and suppression of premature labor [18]–[21]. Our finding of the potent neuroprotective effects of isoxsuprine hydrochloride in ischemic stroke warrants further investigation.
